# ^18^F-FDG PET/CT显像中肺外病变对肺癌的辅助定性诊断价值

**DOI:** 10.3779/j.issn.1009-3419.2012.02.03

**Published:** 2012-02-20

**Authors:** 宝明 米, 卫星 万, 春景 郁, 徐阳 尤, 峰 蒋, 庆军 游

**Affiliations:** 1 214062 无锡，苏州大学附属第四医院核医学科 Department of Nuclear Medicine, the Fourth Affiliated Hospital of Soochow University, Wuxi 214062, China; 2 210009 南京，南京医科大学附属江苏省肿瘤医院胸外科 Department of Thoracic Surgery, Nanjing Medical University Affiliated Cancer Hospital of Jiangsu Province, Nanjing 210009, China; 3 214062 无锡，苏州大学附属第四医院胸外科 Department of Thoracic Surgery, the Fourth Affiliated Hospital of Soochow University, Wuxi 214062, China

**Keywords:** 肺肿瘤, 体层摄影术, 发射型计算机, X线计算机断层术, 诊断, Lung neoplasms, Tomography, Emission-computed tomography, X-ray computed tomography, Diagnosis

## Abstract

**背景与目的:**

^18^F-脱氧葡萄糖（fluorodeoxyglucose, FDG）正电子发射体层摄影术/计算机体层摄影术（positron emission tomography/computed tomography, PET/CT）对肺癌和部分肺良性病变的鉴别诊断仍有一定困难，本研究旨在探讨PET/CT显像中肺外病变对肺癌的辅助定性诊断价值。

**方法:**

回顾性分析126例行PET/CT检查的疑诊肺癌病例。初始诊断仅根据肺内病变的PET表现、平均标准摄取值（mean standardized uptake value, SUVmean）和CT征象，然后根据肺外病变情况对肺内病变的诊断进行修正，比较修正前后诊断结果有无差异。

**结果:**

PET/CT发现81例同时伴有肺外病变，肺外转移性病变使13例可疑恶性修正为肯定恶性，1例良性修正为肯定恶性；肺外非转移性病变使2例可疑恶性修正为肯定恶性，1例可疑恶性修正为良性。除2例全身结核被错误修正为恶性外，其它15例经病理证实均为正确修正。修正前后PET/CT的诊断结果差异有统计学意义，修正诊断率为13.5%（17/126），修正诊断正确率为88.2%（15/17）。

**结论:**

肺外病变的发现对肺癌的术前定性有着较好的实用价值，提高了诊断效能，但仍然要注意与全身多发结核等炎性病变鉴别。

## Abstract (en)

**Background and objective:**

^18^F-FDG PET/CT has several shortcomings in discriminating between lung carcinoma and pulmonary benign lesions. The aims of the present study is to explore the value of extra-lung lesions on ^18^F-FDG PET/CT image in the diagnosis of lung cancer.

**Methods:**

A total of 126 suspected lung cancer patients underwent ^18^F-FDG PET/CT scan. Preliminary diagnoses were based on the PET characteristics, SUVmean value, and CT characteristics of the lesions in the lung, and the diagnoses were modified based on the detected extra-lung lesions. The difference between the two methods and their disparity were calculated.

**Results:**

Extra-lung lesions were identified on the PET/CT image in 81 patients; extra-lung metastasis modified 13 probable malignancies to affirmative malignancy and 1 probable malignancy to benign lesion. Non-metastasis modified 2 probable malignancies to affirmative malignancy and 1 probable malignancy to benign lesion. Fifteen were correct, whereas 2 were misdiagnosed. The diagnoses modification rate was 13.5% (17/126), and the modified diagnoses accuracy is 88.2% (15/17).

**Conclusion:**

Extra-lung lesions demonstrated on ^18^F-FDG PET/CT improved the diagnostic accuracy of lung cancer. Tuberculosis was identified as the most important reason for false positive diagnoses after modification by extra-lung lesions.

肺癌的定性诊断一直是影像工作的重点也是难点，能够显示病灶代谢特征的分子影像技术正电子发射体层摄影术和正电子发射体层摄影术/计算机体层摄影术（positron emission tomography/computed tomography, PET/CT）对提高肺癌的诊断效能取得了良好效果^[1, 2]^。但是，由于目前用于PET显像的常规放射性药物^18^F-脱氧葡萄糖（fluorodeoxyglucose, FDG）在恶性病变和炎性病变中的浓聚程度有部分交叉，而且各种病理类型的肺癌生物学特征有一定差异，导致PET/CT对部分病例难以作出明确定性诊断，特别是PET和CT两种显像结果矛盾的病例。由于PET/CT显像可以反映全身多个器官的情况，所以在PET/CT诊断过程中往往可根据肺外其它器官病灶的显像结果对肺部占位进行辅助定性，但对其实际的诊断价值以及是否仍然存在假阳性或假阴性等问题的相关文献报道较少，本研究主要探讨此问题。

## 1 资料与方法

### 1.1 一般资料

收集2010年2月-2011年8月苏州大学附属第四医院PET/CT中心因疑诊肺癌行^18^F-FDG PET/CT检查的患者126例，显像前均未经相关治疗。患者包括男性78例，女性48例，年龄23岁-85岁，平均60.99岁。肿块最大径范围为1.0 cm-9.3 cm。所有的病变性质由病理、影像及临床随访证实，随访时间为2个月-18个月。经手术、活检、细胞学检查等确诊恶性114例，其中经术后病理确诊腺癌55例，鳞癌18例，癌肉瘤1例，肉瘤样癌1例，小细胞肺癌7例，神经内分泌癌2例，肺粘膜相关组织淋巴瘤1例，29例经病理或细胞学等检查到癌细胞，未明确病理分型；良性12例，其中炎性假瘤2例，炎性肉芽肿3例，炎症2例，结核4例，结节病1例。肺外部变均经多种影像学检查及临床随访诊实。

### 1.2 ^18^F-FDG PET/CT图像采集

使用Siemens Biograph 64 HD PET/CT扫描仪。检查前患者禁食6 h以上，血糖水平＜150 mg/mL，静息、平卧15 min后注射示踪剂^18^F-FDG，注射剂量0.15 mCi/kg。静卧60 min后行体部和头部PET/CT扫描，扫描范围从颅顶至股骨上段。CT扫描参数为120 kV，40 mAs-100 mAs（根据体重），扫描层厚5 mm，进床速度5 mm/床位，重建层厚0.625 mm-5 mm。PET扫描参数为2 min/床位，3D采集，采用有序子集最大期望值法图像重建，显示矩阵为128×128，图像层厚为5 mm，以冠状、矢状、横断进行图像显示及两种图像融合。因呼吸等原因所造成的两种图像不匹配者采取人工视觉调整校正，较严重者进行重新胸部扫描。

### 1.3 PET/CT图像分析

由两位具有2年以上PET/CT工作经验的核医学医师进行盲法影像判读，对于不同意见者协商决定。PET/CT图像分为肺部原发病变单独分析和肺内病变加肺外病变综合分析。肺部原发病变根据PET和CT图像特征判断：PET图像示病灶呈肿块状、不规则或结节状异常放射性浓聚，平均标准摄取值（mean standardized uptake value, SUVmean）≥2.5为阳性，CT图像示病灶具有分叶、毛刺、小泡征、胸膜凹陷征和支气管血管集束征等形态学恶性征象者为阳性。PET和CT图像均为阳性则肺部原发病变判断为肯定恶性，有其一则判断为可疑恶性，均为阴性则判断为良性。

肺外病变：PET图像示肺外高于周围正常组织的放射性浓聚，对应CT伴或不伴形态和密度学改变，排除棕色脂肪、肠道、肌肉、女性卵巢等生理性摄取和一些炎性淋巴结的摄取（炎性淋巴结判断标准：放射性摄取程度较轻，SUVmean＜2.5，对应CT示淋巴结最大短径＜1cm，随访1年未发现肿瘤性疾病）即为阳性；排除原发恶性病变和一些累及多器官的炎性非肿瘤性病变，如结核、自身免疫性病变等（结核病变以肺部典型的浸润性或空洞等CT影像学征象判断，自身免疫性病变PET图像可见多发小淋巴结放射性摄取轻度增高，多个关节放射性摄取轻度增高，脾脏、全身骨髓等的弥漫性放射性摄取增高）即为转移。根据肺外病变对肺部病变性质进行修正：肺外病变为转移者则肺部病变修正为肯定恶性；肺外病变为非转移病变，则视具体情况用于肺部恶性病变的排除。

### 1.4 统计学方法

以病理、影像和临床随访结果为最终标准，分别统计分析以肺部原发病变单独诊断和联合肺外病变诊断结果，比较两种方法定性诊断差异。采用SPSS 19.0统计分析软件包对两种方法的诊断结果进行χ^2^检验。*P*＜0.05为差异有统计学意义。

## 2 结果

### 2.1 肺外病变对肺原发病变诊断的修正结果

肺的原发病变中，PET阳性114例，阴性12例；CT阳性104例，阴性22例。81例同时伴有肺外发现，其中68例提示淋巴结转移，11例提示肝转移，4例腹膜转移，23例骨转移，胸膜转移14例，8例肾上腺转移，5例脑转移，2例心包转移，脊髓和腹膜、肌肉等少见部位转移4例；3例为非转移性病变（结核2例，结节病1例）。综合PET和CT两种结果，仅根据肺原发病变，96例判断为肯定恶性，26例为可疑恶性，4例为良性。通过肺部原发病变联合肺外病变对17例进行了修正诊断，其中肺外转移性病变使13例可疑恶性修正为肯定恶性（图 1），1例良性修正为肯定恶性（图 2）；肺外非转移性病变使2例可疑恶性修正为肯定恶性，1例可疑恶性修正为良性（图 3）。修正后肯定恶性、可疑恶性和良性分别为112例、10例和4例。肺外病变对肺原发病变诊断的修正结果比较见表 1。

**1:**
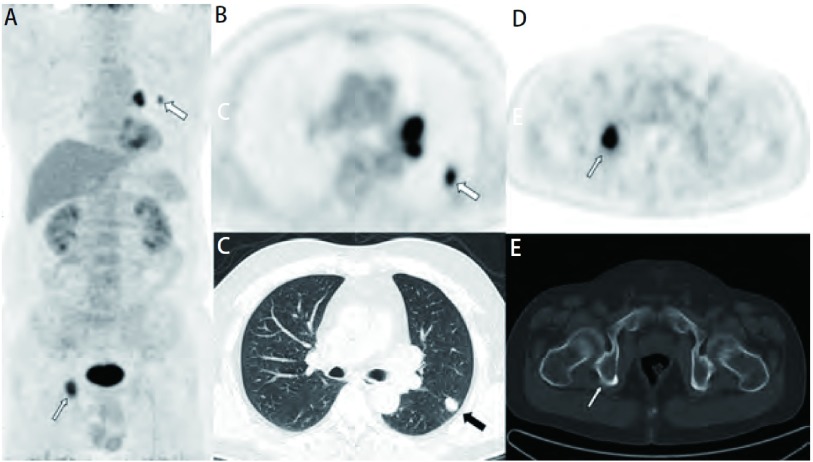
男，52岁，左肺一枚结节性病灶，CT未见典型恶性征象，PET示异常浓聚（A、B、C，粗箭头），SUVmean：3.5，按照诊断标准初步诊断为可疑恶性，但全身PET/CT发现该病例伴有同侧肺门肿大淋巴结和右侧坐骨的团块状异常FDG浓聚（A、D、E，细箭头），修正诊断为肯定恶性，最终经活检证实为小细胞肺癌。 A male patient, 52 years old, CT demonstrates a nodule in his left lung but not seemed as malignancy. A hypermetabolic lesion with SUVmean value as 3.5 was identified on PET image (A, B, C, crude arrow). Preliminary diagnosed as probable malignancy. Whole body PET/CT showed FDG accumulate in ipsilateral hilar lymph nodes and right ischium (A, D, E, fine arrow). Modified diagnosis as affirmative malignancy, and ultimately confirmed by biopsy of small cell lung cancer.

**2:**
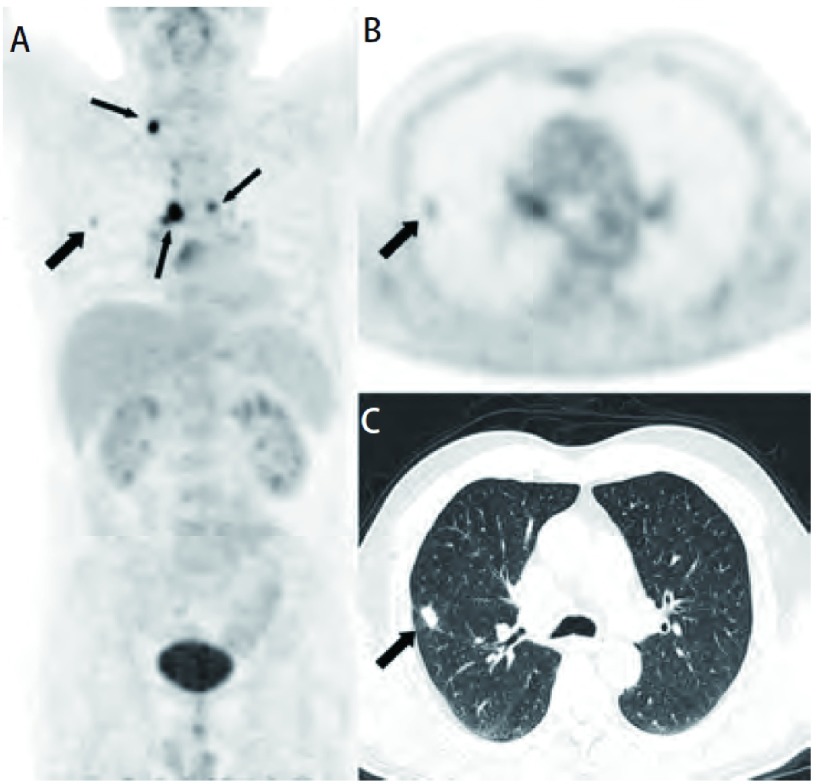
男，63岁，右肺发现结节状病灶，CT无典型恶性征象，PET图像仅有轻度FDG浓聚（A、B、C，粗箭头），SUVmean：2.17，按照诊断标准初步诊断为良性病变，根据同侧肺门肿大淋巴结和纵隔、右侧锁骨上淋巴结均FDG代谢异常浓聚，修正诊断为肯定恶性。术后免疫组化证实为腺癌。 A male patient, 63 years old, CT demonstrates a nodule in his right lung but without typical malignant signs. PET image showed only mild uptake with SUVmean value as 2.17 (A, B, C, crude arrow). Preliminary diagnosed as benign lesion. Whole body PET/CT showed FDG accumulate in bilateral hilar lymph nodes and right supraclavicular lymph node (A, fine arrow). Modify diagnosis as affirmative malignancy. Immunohistochemistry confirmed as adenocarcinoma.

**3:**
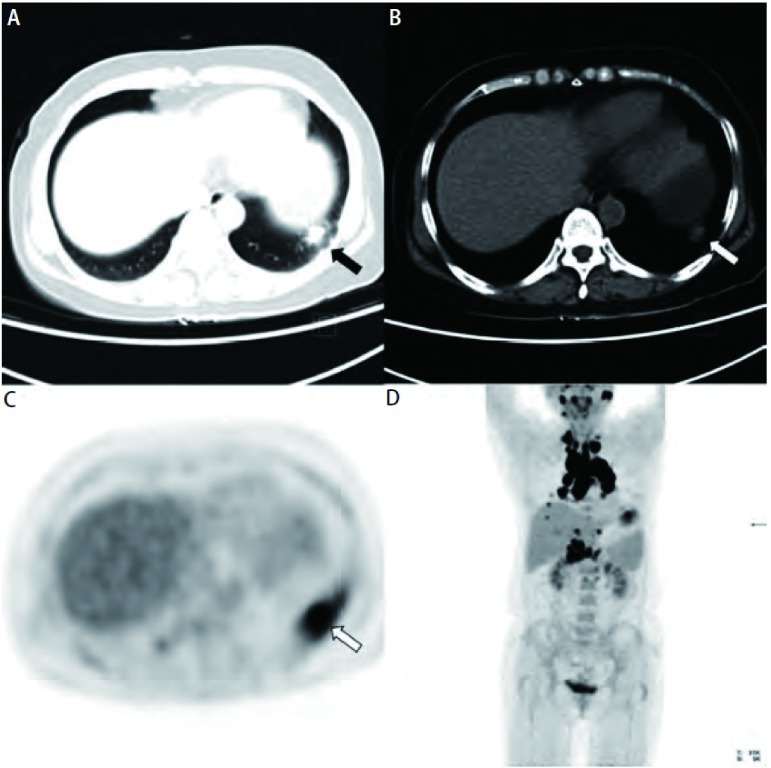
女，53岁，左肺下叶一枚结节性病灶，CT未见典型恶性征象（A、B），PET示异常浓聚（C），SUVmean: 8.07，按照肺内病灶初步诊断为可疑恶性，但全身PET/CT发现该病例双侧肺门肿大、纵隔、双侧颈部、腹膜后等部位的肿大淋巴结均呈FDG异常浓聚（D），根据其肺门淋巴结的“八字型”征象将诊断修正为结节病，最终经活检证实。 A female patient, 53 years old, CT demonstrates a nodule in his left lung but not seemed as malignancy (A, B, crude arrow). A hypermetabolic lesion with SUVmean value as 8.07 was identified on PET image (C, crude arrow). Preliminary diagnosed as probable malignancy. Whole body PET/CT showed FDG accumulate in bilateral hilar lymph nodes, mediastinal lymph nodes, bilateral cervical lymph nodes and retroperitoneal lymph nodes et al. (D). Modified diagnosis as sarcoidosis according to "八" sigh in hilar nodes, subsequently confirmed by biopsy.

**1:** 肺外病变对肺原发病变诊断的修正结果比较 Comparison between preliminary diagnosis and modified diagnosis

Diagnostic methods	Affirmative malignancy	Probable malignancy	Benign lesions
Preliminary diagnosis	96	26	4
Modified diagnosis	112	10	4

对以肺部原发病变单独诊断和肺内病变联合肺外病变进行诊断两种方法诊断结果进行检验，差异有统计学意义（χ^2^=8.342, *P*＜0.05）。除2例全身结核被错误修正为恶性外，其它15例经病理、影像及临床随访证实均为正确修正。根据肺外病变对肺内占位的修正诊断率为13.5%（17/126），修正诊断正确率为88.2%（15/17）。

### 2.2 修正诊断前后PET/CT诊断准确度指标对比（表 2）

**2:** PET/CT准确度指标对比 Comparison of diagnosis index

Diagnostic methods	Sensitivity	Specificity	Accuracy	Positive predictive value	Negative predictive value
Preliminary diagnosis	82.5%（94/114）	83.3%（10/12）	82.5%（104/126）	97.9%（94/96）	33.3%（10/30）
Modified diagnosis	94.7%（108/114）	66.7%（8/12）	92.1%（112/126）	96.4%（108/112）	57.1%（8/14）

以病理学和最终随访结果为标准相比较，假阴性从20例（15例CT阴性，4例PET阴性，1例两者均为阴性）减为6例，但假阳性由2例上升到4例（3例为结核，1例为炎性肉芽肿）。肺外病变使PET/CT诊断肺癌的灵敏度和准确性得到了提升，而特异性下降。阳性预测值无明显差别，阴性预测值明显增加。

## 3 讨论

### 3.1 仅根据肺部病变诊断的不足

统计表明以诊断肺癌为目的的PET/CT检查占全国各PET/CT中心诊断肿瘤患者的1/4以上^[3]^。目前在日常工作中，各PET/CT中心医生在诊断肺部病灶时往往都分别分析两种影像后进行综合判断。FDG PET对肺部肿块的评价有非常高的价值，Gould等^[4]^通过*meta*分析显示，单纯PET对肺单发结节良恶性鉴别的灵敏度为97%，特异性为78%。但是FDG PET显像在对肺部肿块的定性上仍然存在两个主要问题：①结核和肉芽肿、炎性假瘤等炎性病变由于含有较多的巨噬细胞也可较多的摄取FDG，所以易造成假阳性，本研究中有5例良性病变PET呈阳性表现；②对于＜1.5 cm的肿瘤或肺原位腺癌时，FDG PET有相当高的假阴性率；本研究中有5例肺癌病灶PET呈阴性，均为体积较小的腺癌。肺癌的典型CT形态学征象包括病灶较大、边缘不规则（分叶征、毛刺征）、内部密度不均匀（空泡征、支气管充气征、空洞征、磨玻璃样密度）、对邻近结构的侵犯（与支气管的关系、血管集束征、晕轮征、胸膜凹陷征）等。但是有部分肺癌并无这些特异性征象，而结核等部分良性病变却具有这些征象。本研究中有15例肺癌病例其肺部病变的CT表现不具有典型恶性征象，而有6例良性病例却具有恶性征象。所以，单纯通过肺部病变的PET/CT表现来进行定性诊断对于部分病例有一定困难，本研究114例恶性病例，94例通过肺部表现明确诊断，19例无法明确定性，1例误判为良性；12例良性病例中仅有3例明确诊断为良性，7例无法明确定性，2例误判为恶性。

### 3.2 根据肺外病变对诊断的修正价值

肺癌除了易引起纵隔、锁骨上等部位淋巴结的转移，还特别容易通过血行转移至肝、骨骼、脑、肾上腺等器官。CT、磁共振成像（magnetic resonance imaging, MRI）等显像方法一般仅做一个部位的检查，所以在一些肺原发病灶形态学征象不是很明确的病例时往往较难明确诊断，而PET/CT是一次全身成像，且其灵敏度非常高，所以可以通过全身其它器官的显像结果来辅助判断肺部病变的良恶性质。本研究中通过肺外转移性病变，修正了14例仅根据肺部原发病变所做的初步诊断，使13例可疑恶性和1例良性最终诊断为肯定恶性，对明确诊断起到了很大作用，并且提升了医生的诊断信心。肺癌淋巴结转移的机率非常高，以同侧肺门和纵隔最多，但是转移在颈部、腹腔、腹膜后等远处的病例并不少见。除肺癌以外的多种感染、结核、结节病和反应性增生等病都可以引起多发淋巴结肿大^[5]^，所以通过发现异常淋巴结可以明显提高诊断的灵敏度，但特异性会降低。本研究中29例患者伴有除肺门、纵隔外的淋巴结转移，有13例因发现淋巴结转移而将可疑恶性诊断修正为肯定恶性，其中11例正确。骨骼是肺癌另一个好发转移部位，主要通过血行转移，且以溶骨性病变为主，PET/CT由于既可以显示骨转移病灶代谢异常增高，同时部分病灶在CT上会有骨质结构的改变，所以对肺癌骨转移的检出率较骨扫描、CT扫描高^[6, 7]^。由于骨转移病灶相对较为特异，所以在PET/CT扫描中发现骨转移灶对提高诊断准确性有很大帮助，本研究中5例由于发现骨转移病变正确地将可疑诊断修正为肯定恶性。由于肝脏的良性病变很少浓聚FDG，结核等病变较少累及肝脏，所以PET/CT诊断肝转移的特异性较高，能检查出一些常规影像学不能确定的肝转移，确定和排除常规影像学不能确定的肝转移^[8]^。本研究中共发现肝转移者12例，有2例对诊断修正起到了作用，均为正确修正。肺癌脑转移、肾上腺转移的机率也较高^[9, 10]^，所以脑部和肾上腺转移病灶的发现对肯定肺癌的诊断也有非常大的价值。心包、肌肉等部位的转移机率较少，且多晚于其它部位转移，故这些部位的转移灶发现对诊断的修正价值较小。

肺外转移性病变一方面可以帮助肺癌诊断的确立，同时肺外一些非转移性病变，也有助于肺癌的排除。本研究中1例结节病，肺部一处肿块状病灶FDG代谢异常增高，但其CT征象缺乏典型恶性病变征象，故仅根据肺内PET/CT表现诊断为可疑恶性，但是其全身PET/CT可见颈部、纵隔、双侧肺门、膈脚后、腹膜后多发肿大淋巴结，双侧肺门淋巴结呈较典型的“八”字型征象，故考虑为结节病，最终得到病理确诊。

本研究根据肺外病变对肺内占位的修正诊断率为13.5%（17/126），修正诊断正确率为88.2%（15/17），使这部分病例得到了明确的定性诊断，尽早被安排了治疗计划。

### 3.3 假阳性问题

PET/CT全身显像由于发现了较多的肺外病灶，但这些病灶形态、分布和代谢特点有时并无特异性，所以仍然会造成少量的假阳性问题。由于结核可以在全身多个器官发生，且表现往往较为复杂^[11]^，所以PET/CT显像将全身结核病变误诊为恶性肿瘤多发转移时有发生。本研究中有2例结核病例误诊为恶性病变，就是因为这2例结核病例的病灶特点非常不典型，异常淋巴结遍布全身，骨骼上的结核病灶位于椎体附件等骨转移好发部位，所以造成了误诊。多器官侵犯的结节病也可造起类似的诊断困难，在今后的工作中还要更加注意结合临床病史、血肿瘤标记物等进行详细鉴别。

综上所述，在PET/CT显像中通过对肺外病变的分析对肺癌的辅助定性诊断有着较好的实用价值，提高了诊断效能，提升了医生的诊断信心，但是在实际应用中仍然要注意与全身多发结核等炎性病变的鉴别。由于本组研究病例中良性病变所占比例较少，希望今后在更多病例数量的应用研究中进一步证实此方法的价值。
